# Supplementary material for: Ultra-Sensitive Determination of Cadmium in Food and Water by Flame-AAS after a New Polyvinyl Benzyl Xanthate as an Adsorbent Based Vortex Assisted Dispersive Solid-Phase Microextraction: Multivariate Optimization
Source: Foods. 2023 Sep 28;12(19):3620. doi: 10.3390/foods12193620 (PMC10572459; doi:10.3390/foods12193620)
Supplement: Supplementary file 1 [file foods-12-03620-s001.zip › foods-2578312-supplementary.pdf]

Table S1. Analysis of Variance (ANOVA).

Source	DF	Adj SS	Adj MS	F-Value	P-Value
Model	16	2530.27	158.142	7.52	0.000
Blocks	2	924.34	462.169	21.97	0.000
Linear	4	530.47	132.617	6.30	0.005
A	1	9.38	9.375	0.45	0.516
B	1	219.01	219.010	10.41	0.007
C	1	126.04	126.042	5.99	0.029
D	1	176.04	176.042	8.37	0.013
A*A	1	559.29	559.292	26.58	0.000
B*B	1	369.81	369.810	17.58	0.001
C*C	1	327.06	327.060	15.54	0.002
D*D	1	259.88	259.882	12.35	0.004
A*B	1	0.00	0.000	0.00	1.000
A*C	1	0.00	0.000	0.00	1.000
A*D	1	0.00	0.000	0.00	1.000
B*C	1	0.00	0.000	0.00	1.000
B*D	1	0.00	0.000	0.00	1.000
C*D	1	0.00	0.000	0.00	1.000
Error	13	273.52	21.040		
Lack-of-Fit	10	273.52	27.352		
Pure Error	3	0.00	0.000		
Total	29	2803.79			

Table S2. Effect of some anions and cations on pre-concentration and determination of Cd(II) (100 ng L^{-1}, N=5).

Anions/cations	Tolerable concentration (mg L^{-1})	Recovery (%)	RSD (%)
Ca^{2+}	1500	97±2	3.6
CH_3COO^-	1000	99±4	3.9
Ba^{2+}	1000	97±2	4.1
$SO4^{2-}$	2000	99±3	4.2
HCO_3^-	1000	99±4	4.0
F^-	500	98±5	3.6
Oxalic acid	500	97±3	3.2
CO_3^{2-}	2000	97±4	3.4
Zn^{2+}	50	96±2	2.8
Sn^{4+}	50	97±4	2.6
Ni^{2+}	30	96±3	2.9
Al^{3+}	30	95±4	3.2
Co^{2+}	25	94±5	3.8
Fe^{3+}	50	92±3	4.6
Pb^{2+}	25	92±5	4.3
As^{3+}	25	90±4	4.2
Na^+	5000	95±2	3.7
Cl^-	10000	95±4	4.4

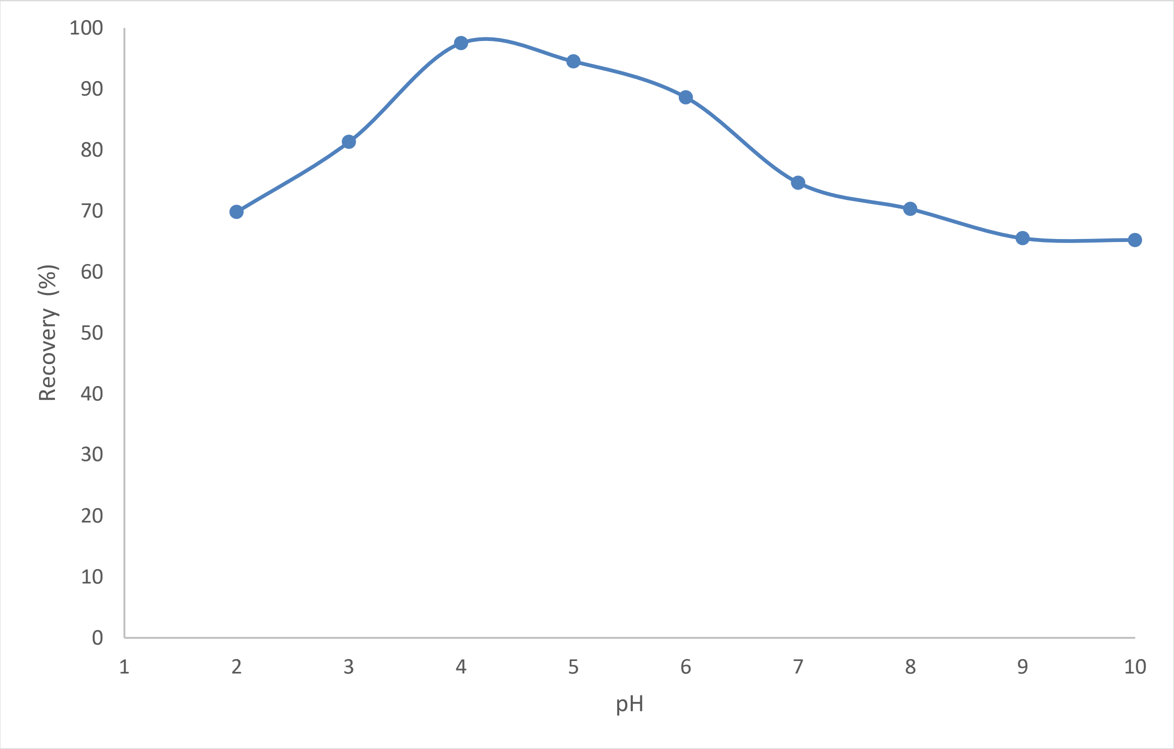

Figure S1. Effects of pH (sorbent amount: 125 mg, mixing type: vortex, mixing time: 7.5 min, solvent type: EtOH, EtOH volume: 1250 μL, eluent time: 120 s, number of resume of sorbent: 10, sample volume: 200 mL).

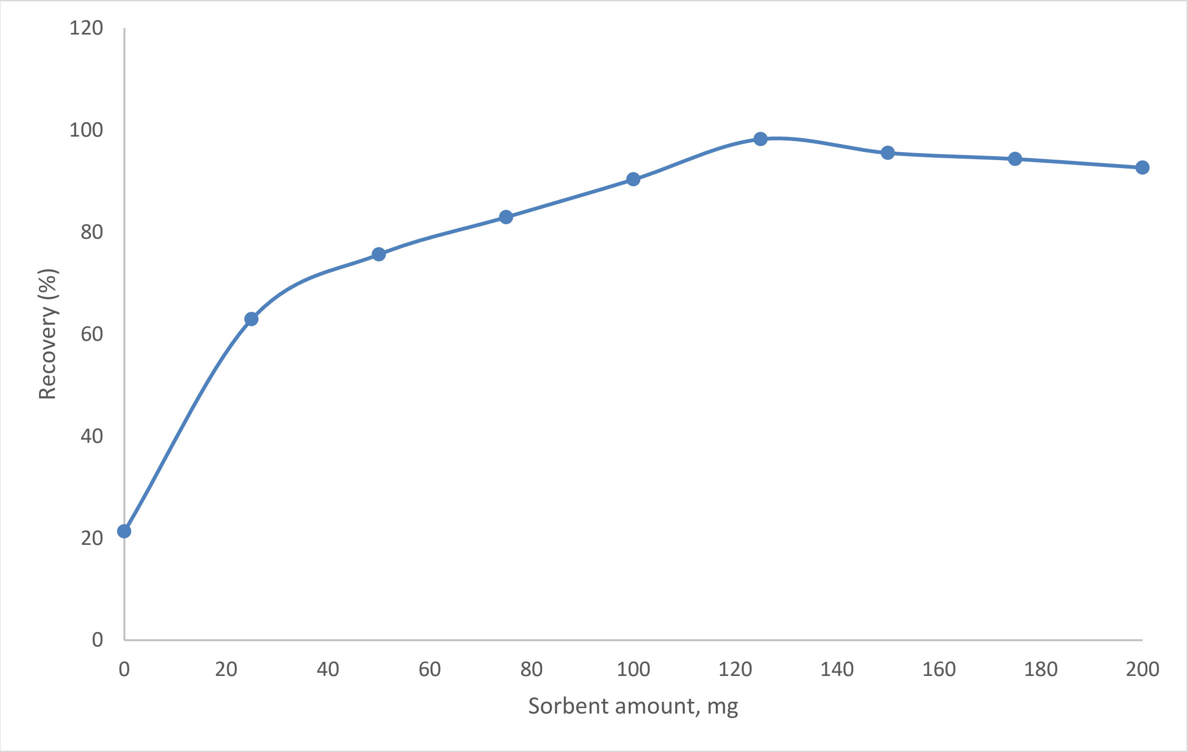

Figure S2. Effects of sorbent amount (pH 4, mixing type: vortex, mixing time: 7.5 min, solvent type: EtOH, EtOH volume: 1250 μL, eluent time: 120 s, number of resume of sorbent: 10, sample volume: 200 mL).

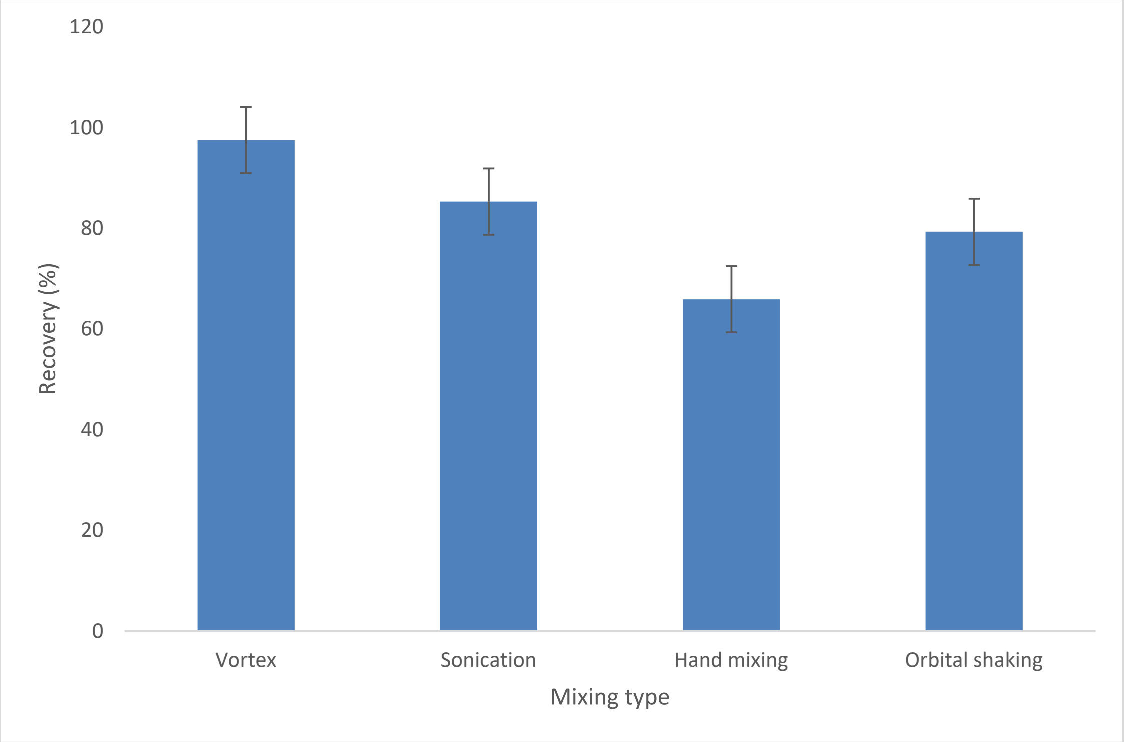

Figure S3. Comparison of mixing type (pH 4, sorbent amount: 125 mg, mixing time: 7.5 min, solvent type: EtOH, EtOH volume: 1250 μL, eluent time: 120 s, number of resume of sorbent: 10, sample volume: 200 mL).

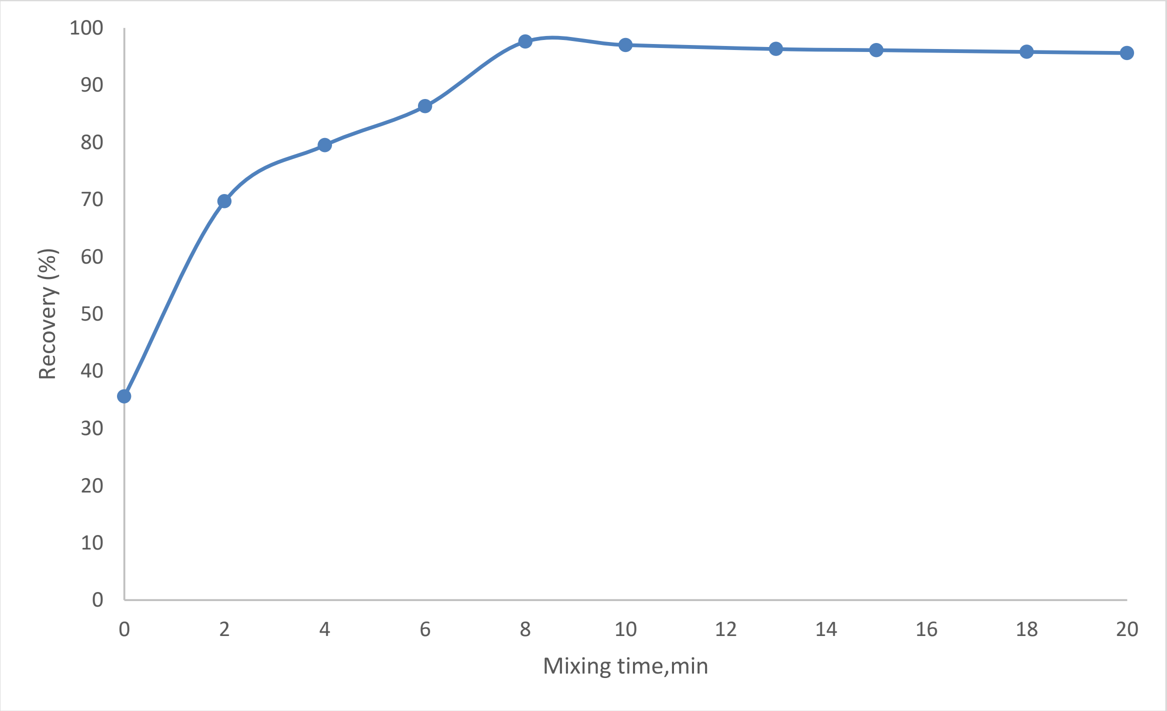

Figure S4. Effect of mixing time (pH 4, sorbent amount: 125 mg, mixing type: vortex, solvent type: EtOH, EtOH volume: 1250 μL, eluent time: 120 s, number of resume of sorbent: 10, sample volume: 200 mL).

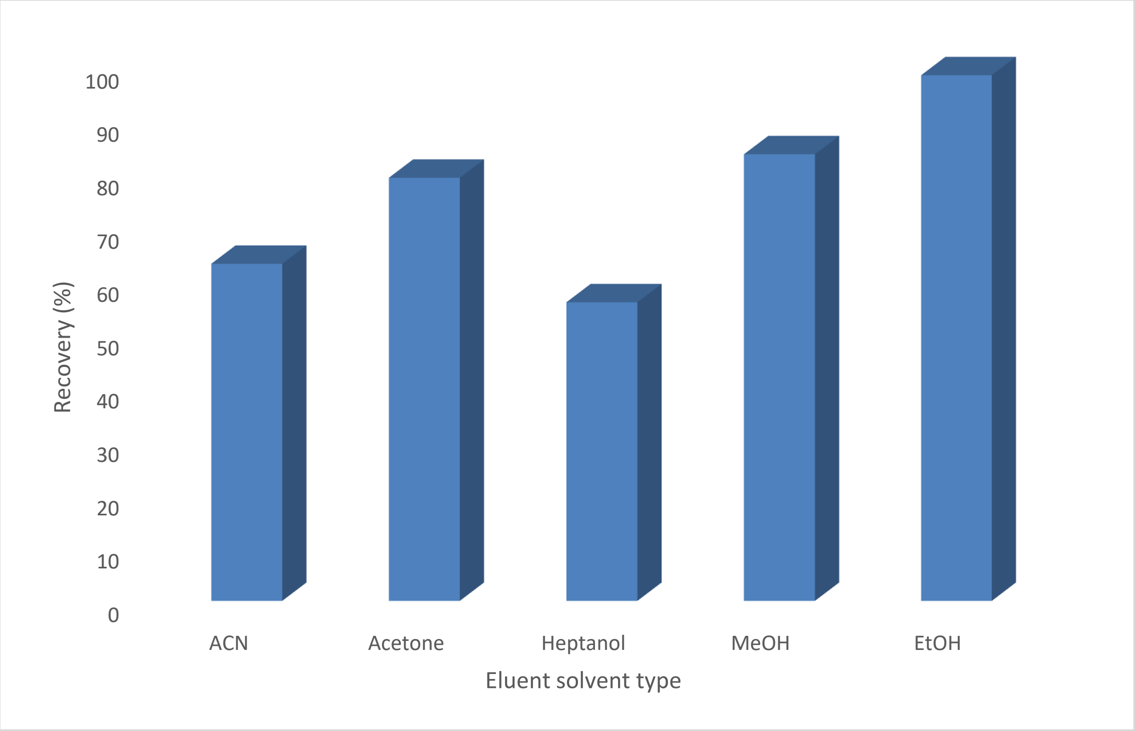

Figure S5. Comparison of different eluent solvent types (pH 4, sorbent amount: 125 mg, mixing time: 7.5 min, mixing type: vortex, EtOH volume: 1250 μL, eluent time: 120 s, number of resume of sorbent: 10, sample volume: 200 mL).

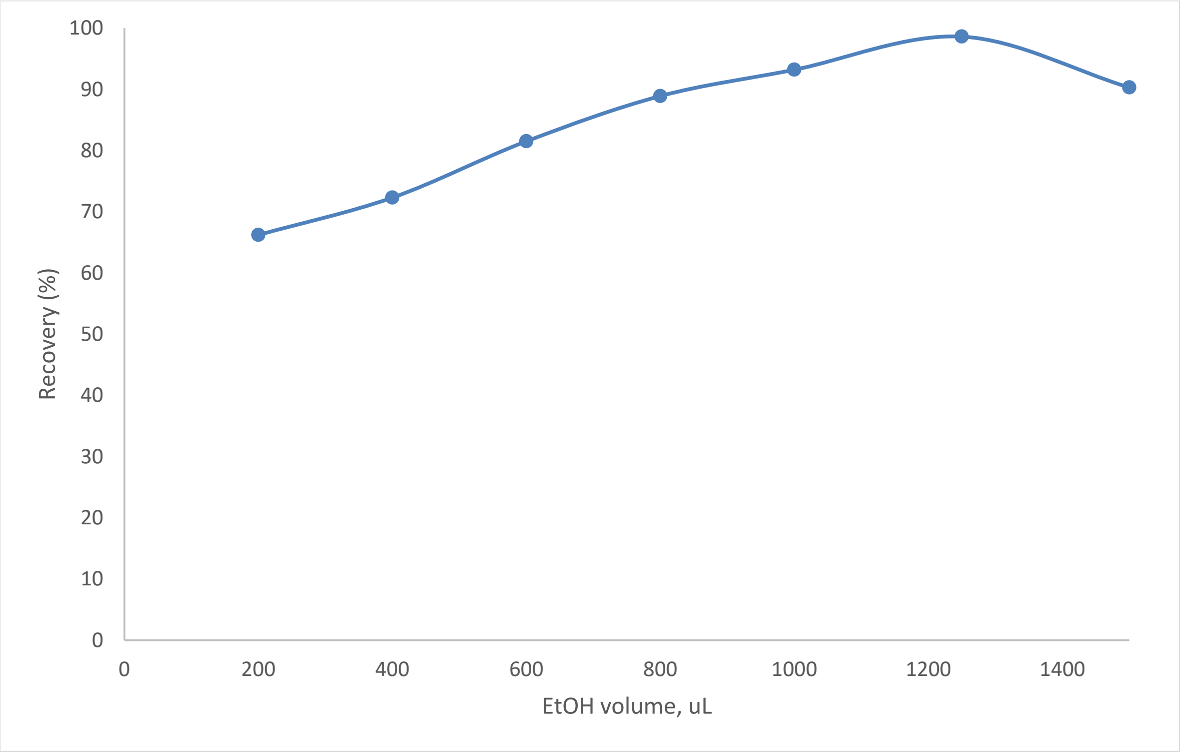

Figure S6. Effects of EtOH volume (pH 4, sorbent amount: 125 mg, mixing time: 7.5 min, mixing type: vortex, eluent time: 120 s, number of resume of sorbent: 10, sample volume: 200 mL).

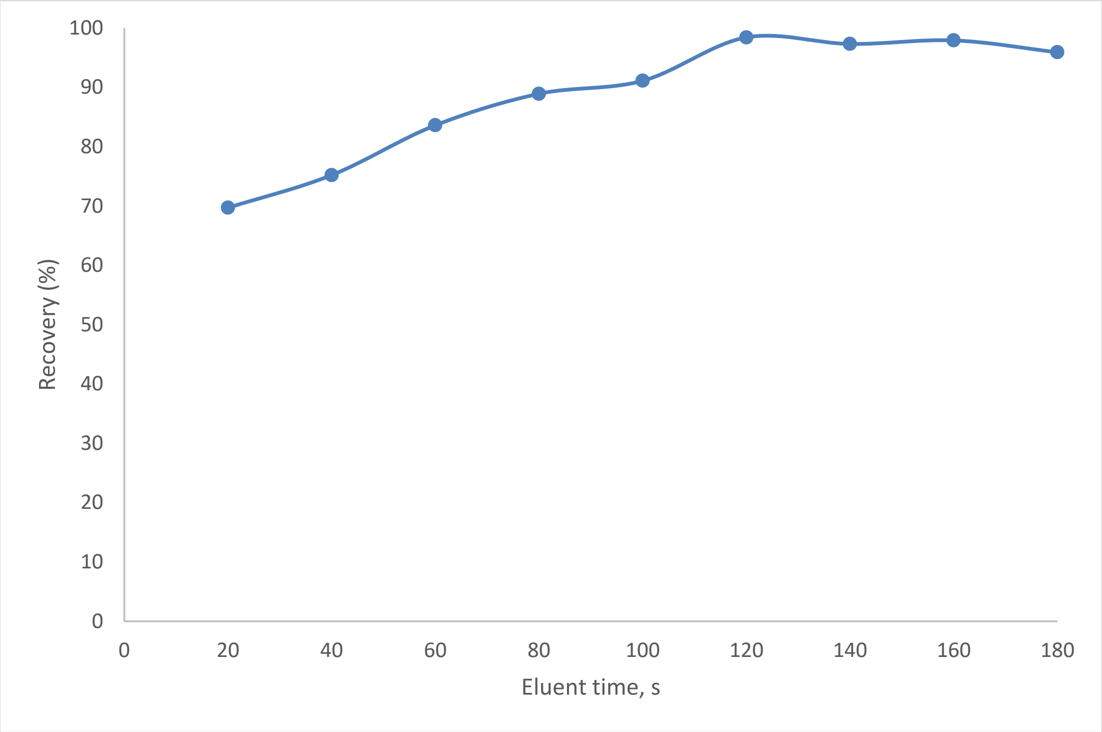

Figure S7. Optimizing the eluent time (pH 4, sorbent amount: 125 mg, mixing type: vortex, mixing time: 7.5 min, eluent time: 120 s, number of resume of sorbent: 10, sample volume: 200 mL).

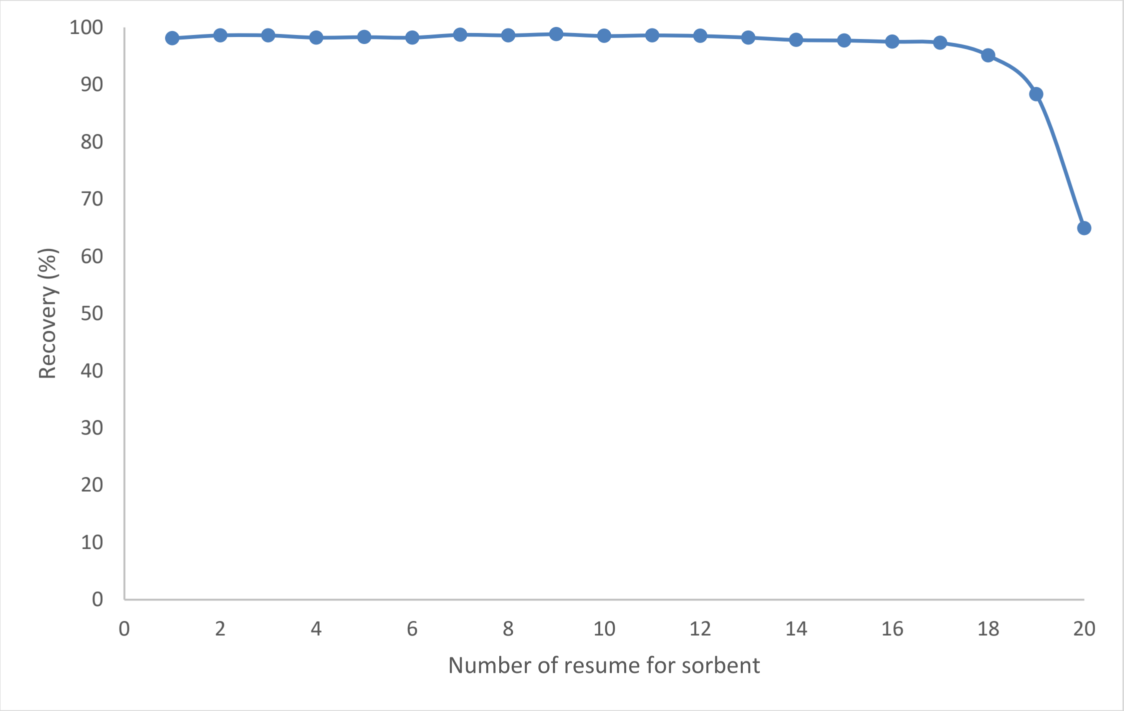

Figure S8. Effects of number of resume for sorbent (pH 4, sorbent amount: 125 mg, mixing type: vortex, mixing time: 7.5 min, eluent time: 120 s, sample volume: 200 mL).

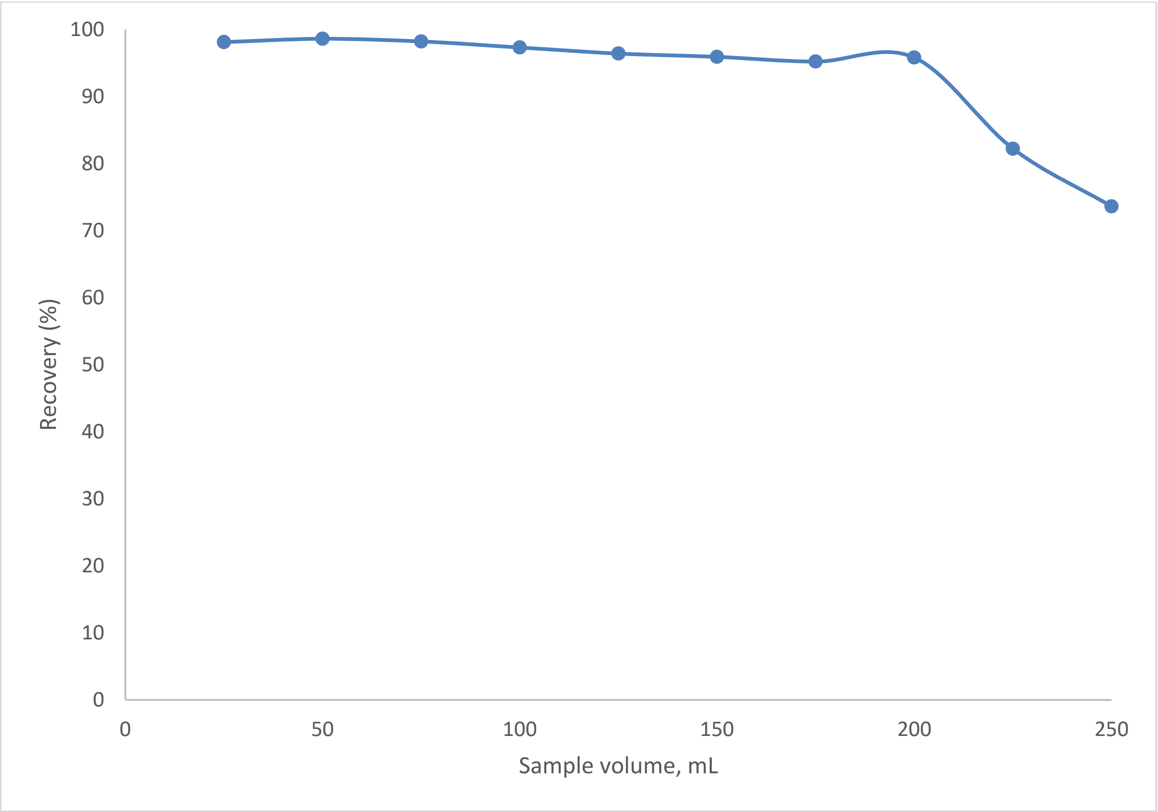

Figure S9. Effects of samples volume (pH 4, sorbent amount: 125 mg, mixing type: vortex, mixing time: 7.5 min, solvent type: EtOH, EtOH volume: 1250 μL, eluent time: 120 s, number of resume of sorbent: 10).
